# Modelling social vulnerability in sub-Saharan West Africa using a geographical information system

**DOI:** 10.4102/jamba.v7i1.155

**Published:** 2015-05-28

**Authors:** Olanrewaju Lawal, Samuel B. Arokoyu

**Affiliations:** 1 Department of Geography and Environmental Management, Centre for Disaster Risk Management and Development Studies, University of Port Harcourt, Nigeria

## Abstract

In recent times, disasters and risk management have gained significant attention, especially with increasing awareness of the risks and increasing impact of natural and other hazards especially in the developing world. Vulnerability, the potential for loss of life or property from disaster, has biophysical or social dimensions. Social vulnerability relates to societal attributes which has negative impacts on disaster outcomes. This study sought to develop a spatially explicit index of social vulnerability, thus addressing the dearth of research in this area in sub-Saharan Africa. Nineteen variables were identified covering various aspects. Descriptive analysis of these variables revealed high heterogeneity across the South West region of Nigeria for both the state and the local government areas (LGAs). Feature identification using correlation analysis identified six important variables. Factor analysis identified two dimensions, namely accessibility and socioeconomic conditions, from this subset. A social vulnerability index (SoVI) showed that Ondo and Ekiti have more vulnerable LGAs than other states in the region. About 50% of the LGAs in Osun and Ogun have a relatively low social vulnerability. Distribution of the SoVI shows that there are great differences within states as well as across regions. Scores of population density, disability and poverty have a high margin of error in relation to mean state scores. The study showed that with a geographical information system there are opportunities to model social vulnerability and monitor its evolution and dynamics across the continent.

## Introduction

Hazards are the precursors of disasters, but all hazards do not need to develop into disasters. How humans respond to everpresent hazards determine whether they will become disasters. Managing disasters require an understanding of risks, hazards, vulnerability and the resources available to minimise the effect of hazards. The relationship ‘risk = hazard * vulnerability’ is common, but there are others (e.g. Flanagan *et al*. 2011) who propose a relationship such as ‘risk = hazard * [*vulnerability – resources*]’, where risk is the likelihood of loss, hazard is a condition likely to cause harm, vulnerability is the extent to which persons or things are likely to be affected, and resources are assets in place that will mitigate the effects of hazards.

Vulnerability implies the potential for loss to either life or property from disaster or hazard events (Van Zyl 2006). Places and people could be vulnerable as a result of biophysical or social attributes (Cutter 1996). Biophysical vulnerability relates to attributes of events and the physical conditions which influence the potential for losses and the ability to recover. Social vulnerability (SoV) relates to the attributes of the society which could impact negatively the outcome of disasters or hazard events. Social inequalities, poverty and various other factors could make people and places susceptible to harm and also hinder their ability to respond to signs and warnings and cope with the consequences of disasters. Characteristics of places where people live could make them susceptible, for example the characteristics of the communities either in relation to access to jobs, transportation links and level of urbanisation could influence social vulnerability. Therefore, we can regard social vulnerability as the factor which could moderate risk and is tied to the social fabric of a place. Social fabric could include community experience with hazard, ability to respond, cope, recover and adapt – all of which are influenced by the housing, economic and demographic attributes of the place (Cutter, Boruff & Shirley 2003).

Currently, there is no literature that has quantified or explored the social vulnerability of people across Nigeria. This could be attributed to the complexity of this phenomenon. An extensive body of work exists across the world in the area of biophysical vulnerability to climate change and other hazards. The exploration of how hazard may impact on people is very important, but the understanding of how and where socially vulnerable people may be affected could positively improve allocation of resources in disaster management as well as social and economic development. This study therefore seeks to address social vulnerability in Nigeria with the objective of contributing to improvement in disaster management in the country.

The purpose of the study is to develop a model of social vulnerability using a geographical information system (GIS), thereby making otherwise inaccessible data available to disaster managers and decision makers across relevant agencies and organisations across the country. The study will identify relevant data and model social vulnerability in Nigeria.

In recent times, disasters and risk management have gained significant attention, especially with increasing awareness of the risk posed by climate change as well as the increasing impact of natural hazards in the developing world. These events further revealed the significance of human decision on the outcome and impact of any hazard events. The increasing urgency of the need to study and develop capacity in the developing world was further highlighted by the work of Jha *et al*. (2010). In their comparison of the level of development and fatalities from disaster, they showed that about 66% of fatalities for the period 1999–2005 occurred in developing nations, followed by least developed countries (26%). This is in agreement with the work of Smith (1992).

Furthermore, data have also revealed an increase in the cost of damages caused by disasters as well as in the number of reported natural and technological disasters (Coppola 2011a). Moreover, it has been reported that the consequences of disaster are likely to be more devastating in the developing world as a result of the interplay amongst population growth, land pressure, economic growth, technological innovations, social expectations and growing interdependence across the globe. It has become extremely important for emerging economies such as Nigeria and its agencies and authorities to understand where potentially vulnerable people are located in order to mitigate the impacts of disaster on them.

The development of a spatially explicit vulnerability model will enhance the ability of decision makers to determine which areas are more vulnerable to different types of risks. The model will provide a useful tool across the four phases of the disaster cycle. Furthermore, there will be an opportunity to track social vulnerability data and analyse those over time, thus allowing better understanding of risk and vulnerability in any given area of the country.

## Methodology

### Study area and data

**Figure 1:**
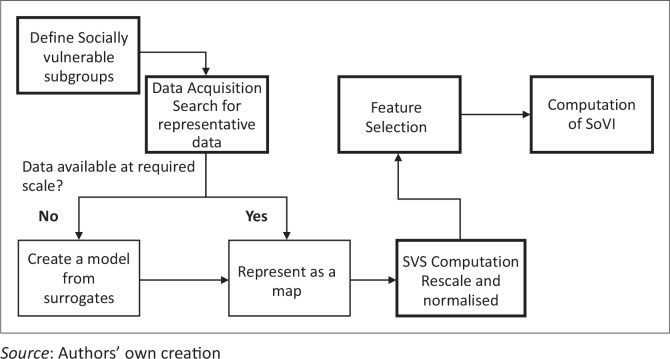
Conceptual model for geographical information system-based social vulnerability index development.

The South West Geopolitical Zone (SWGPZ) of Nigeria is made up of six states, namely Ekiti, Lagos, Ogun, Ondo, Osun and Oyo (Figure 1). This region has a total of 137 local government area (LGAs), which is the lowest governmental authority.

According to the Federal Ministry of Information (FMI) (2012), Ogun has six major ethnic groups, namely the Egba, Ijebu, Remo, Egbada, Awori and Egun. Whilst Yoruba is spoken by the majority of the people, there are a number of dialects across the state. The main cash crops in the state are cocoa, kola nut, rubber, palm oil and palm kernel (FMI 2012). Mineral resources in the state include chalk and phosphate. Ekiti's people are mainly engaged in agriculture, with significant production of cocoa, coffee, plantain, banana and palm oil (FMI 2012). A Yoruba dialect called Ekiti is mainly spoken. Lagos is a major economic centre for the country, with a wide variety of businesses and industries. The Port of Lagos is the main Nigerian port. Ekiti has a mixture of different ethnic groups from across the country, including the Ijebus, Eguns, Aworis and Ekos (pioneer settlers), as well as other Nigerians and foreigners (FMI 2012). Ondo is home to Yoruba sub-ethnic groups such as the Akoko, Akure, Ikale, Ilahe, Ondo and Owo, as well as other minorities such as the Ijaw and Apoi. Agricultural production is significant for the state, with the production of cocoa, coffee, cashew and rubber. Mineral resources found in the state include kaolin, quartzite, limestone and marble. Similarly, Osun people are mainly Yorubas from sub-ethnic groups such as the Osuns, Ifes, Ijesas and Igbominas, each with their own dialect. It is also a mainly agrarian economy with a large production of food and cash crops, similar to other states in the region. Oyo is made up of Yoruba sub-ethnic groups such as the Ibadans, Ibarapas, Oyos, Oke-Oguns and Ogbomoshos. Economic activities and agricultural production are similar to that of Osun, Ondo, Ogun and Ekiti.

The study adapted the method developed by Cutter *et al*. (2003), who suggest that a social vulnerability index (SoVI) quantitatively describes the relative vulnerability of a place based on socioeconomic variables. This index was constructed using data for domains such as socioeconomic status, household composition and disability, minority status and language, and housing and transportation.

A challenge for this study was to find an exact analogue of these SoVI components. Therefore, local and international data sources were explored to identify input variables that had the closest logical fit to the SoVI components. Selected variables (Table 1) were evaluated based on the boundaries for which they were collected and their relevance to the index construction. In determining the location of vulnerable people, it is pertinent that geographic scale is sufficient to differentiate amongst places. However, in the case of Nigeria, where the smallest unit for data collection is the LGA, the study would seek to show differences at such level.

**Table 1:** Domain and selected variables for social vulnerability index construction.

Domains	Selected variables
Socioeconomic status	Poverty (NPOV)§ and extreme poverty (EPOV)§
	Access to improved water –% of population (IWAT)†
	Access to improved sanitation –% of population (ISAN)†
	Electricity in household –% of population (ELECP)†
	Radio in household –% of population (RADP)†
	Television in household –% of population (TVP)†
Household composition and disability	Number of births (NBTH)§
	Number of pregnant women (NPREG)§
	Population of females (FMLE)‡
	Population of 14-year-olds and below (BLW14)‡
	Population of 65-year-olds and above (ABV65)‡
	Population of persons with disability (PWDS)¶
Minority status and language	Net primary attendance rate (NPAR)†
	Net secondary attendance rate (NSAR)†
	Literacy rate – 15 and over (LIT15)†
Housing and transportation	Population density (POPD)§
	Road density (RDACS)††
	Car ownership (COWN)‡‡

Note: Please see the full reference list of the article, Lawal, O. & Arokoyu, S.B., 2015, ‘Modelling social vulnerability in sub-Saharan West Africa using a geographical information system’, *Jàmbá: Journal of Disaster Risk Studies* 7(1), Art. #155, x pages. http://dx.doi.org/10.4102/jamba.v7i1.155, for more information.

†, National Population Commission 2010a; ‡, National Population Commission 2010b; §, GeoData Institute (nd); ¶, Federal Ministry of Women Affairs and Social Development 2011; ††, Socioeconomic Data and Applications Center 2013; ‡‡, World Bank (2007).

The study extracted and collated data for the following four domains:

**Socioeconomic status:** With regard to socioeconomic status, variables relating to the percentage of people in poverty and extreme poverty were selected. In addition, variables relating to access to electricity, improved water, sanitation and mass media were also selected. According to the UNDP (1998), there is a direct connection between poverty and disaster. Therefore, knowledge and understanding of poverty and socioeconomic characteristics of communities play a significant role in disaster risk management and risk reduction.

Poverty and related socioeconomic factors have been found to influence risk perception, behaviour, access and opportunity. For example, poverty often leads to the development of large populations in high risk areas with little or no protection from imminent dangers within such landscape (often the case in shanty towns in many developing countries). These landscapes are usually a last resort and the risk is often known by the socioeconomically disadvantaged inhabitants. Habitation of such environments often leads to further degradation of the environment, further exacerbating the vulnerability of human communities found in such areas. According to the International Federation of Red Cross/Red Crescent Societies (IFRC 2000), extreme poverty limits the capacity to engage in conventional risk reduction measures. This could be attributed to the likely inaccessibility of asset and income to engage in preparation and recovery from disaster (Cutter *et al*. 2003; Morrow 1999). Therefore, when assessing disaster risks, communicating risk or preparing risk communication messages, risk managers should consider the socioeconomic circumstances of the populace.

**Household composition and disability:** Dependent children (below 18 years of age), the elderly (above 65 years of age), individuals with disability and female members of any communities have been reported to be more vulnerable than others in disaster situations (Cutter *et al*. 2003; Morrow 1999; Tierney 2006). This could be attributed to their increased need for assistance during hazard events; being more prone to distress; threat to reproductive health (especially women with stress and psychological problems contributing to fertility and reproductive health issues); lack of access to economic means and power, assets and experience to protect themselves or cope with the situation; and generally lesser ability to recover quickly after disaster.

Moreover, members of households or neighbours of elders living alone may not be able to cope or may be overwhelmed (by helping themselves as well as the elderly) in crisis or emergency situations. In addition to these members of the community, pregnant women and newborns are also very vulnerable. They could increase the vulnerability of the households in which they are present as they share some of the characteristics of the community members mentioned above, such as less ability to recover quickly and lack of life experience to cope with the situation.

**Minority and language dimension:** A literacy-related dataset was selected as it could be used to represent how people interact with risk communication. Literacy and education are major obstacles in disaster risk management, especially in risk communication/public education (Coppola 2011b). They can severely limit the methods available, understanding of risk information or statistics as well as warnings and instructions. Therefore, it is pertinent that information on literacy level be understood and incorporated into social vulnerability assessment. Although the relationship between education and vulnerability is not well understood, there are clear linkages amongst education, income and poverty (Flanagan *et al*. 2011). Moreover, there is a higher likelihood of individuals or households with higher levels of education to access and act on hazard and risk information (Tierney 2006).

Minorities are especially vulnerable, as minority status could bring about social exclusion, which could further exacerbate vulnerability. The only dataset which could be identified is that by Otite (1990), cited in Mustapha (2004). However, this is dated and only shows sums of ethnic minority across regions, making it impossible to disaggregate it for use in this study. The assumption therefore is that data on socioeconomic status could already contain this aspect of social vulnerability.

**Housing and transportation:** Crowding and very high population density has serious implications for quality of life and also poses a high risk for people in crisis and emergency situations. In many developing countries, the quality of housing units is often questionable (e.g. Onuba 2014). When this is coupled with the lack of adequate infrastructure in many urban areas, there is a higher level of vulnerability when compared to less dense landscapes. One can conclude that concentration of population also concentrates the risks for humans. Transportation and social exclusion are intimately linked (Kilroy McGue 2007; 2011). Transportation links and access for low-income household neighbourhoods and their job locations are often bad, which further exacerbate their economic condition and subsequently negatively impact on their vulnerability. Therefore, it is common to find the socioeconomically disadvantaged living in poorly constructed houses which are susceptible to almost every class of hazard (Tierney 2006).

Furthermore, various studies (e.g. Lawal 2009) have highlighted the importance of transportation in influencing land use change and human development, which lends credence to the inclusion of transportation in the modelling of social vulnerability. Transportation influences the direction of change in land use which could lead to increase or decrease in social vulnerability. The poor design of transportation networks can result in fragmentation and isolation of communities. McGue (2011), who reiterates the role of transportation in social vulnerability, cites Cardoso (2008) who argues that the transit system is a factor that could either reinforce or weaken social exclusion and inherent social vulnerability.

Road density can provide an understanding of transportation network accessibility. Higher road density often indicates better accessibility of places, that is, a high proportion of the population will have access to different areas. This also implies that, in case of emergency evacuation, people can be easily moved out and people can be reached for delivery of aid and relief materials. Moreover, car ownership could also be seen as a way of increasing accessibility as well as defining level of personal wealth. Therefore, one could deduce that increasing population density has a negative implication for disaster management (more people competing for limited resources) especially in developing countries, and that higher road density (accessibility) and car ownership can alleviate such problems.

### Method

**Figure 2:**
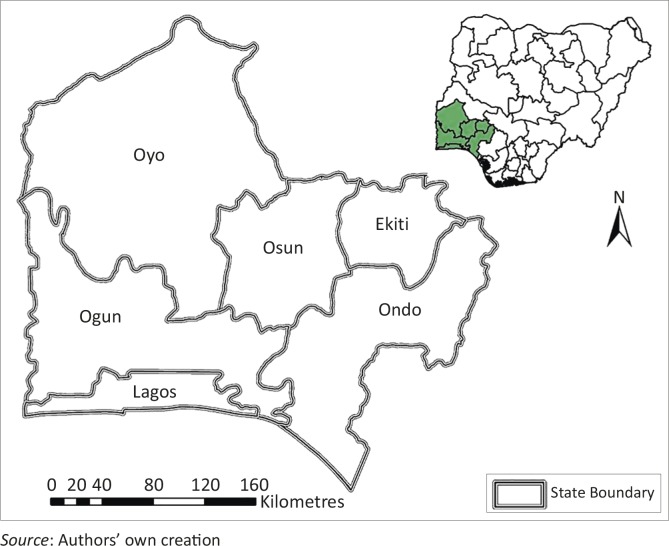
Map showing the location of states within the South West Geopolitical Zone in Nigeria.

The modelling operation followed five steps, namely vulnerable group identification, data acquisition, calculation of social vulnerability scores (SVS), feature selection and derivation of SoVI (Figure 2). Vulnerable subgroup identification involves the review of literature and exploration of data sources to identify factors which have been reported to contribute to vulnerability. The variables selected represent the minimum threshold of data that are necessary to produce a SoVI. Data acquisition followed the identification of vulnerable groups. Datasets obtained were cleaned up and the verification of geographical reference was carried out after which data were processed within GIS and SVS were computed. During the feature selection process, we removed redundant variables and identified dimensions within the remaining dataset. On the basis of the remaining subset of variables, the SoVI map was generated and classified using percentiles to indicate the vulnerability of each LGA in the SWGPZ.

For an accurate representation of social vulnerability, data were collated for each LGA and represented as a raster dataset. Two governmental divisions, namely state and LGA, were used in this study. The nation is divided into states and states are then subdivided into LGAs. In cases where data are only available at the state level, weighting based on the population density data (obtained from UN estimates (GeoData Institute n.d.) was adopted in modelling the LGA level distribution. For example, assuming for a state the literacy level is 54%, to redistribute we assume that this proportion is found across the entire state and obtain 54% of the total population for each grid cell. This was then summed to the LGA boundaries to obtain LGA level data. Similarly, where there was only a percentage at the LGA level, the same operation was carried out. These operations were carried out within ArcGIS (ESRI 2011). The population density map is available in 100 m grid cells and formed the basic map for redistribution operations. We assumed equal distribution of the variables at state level and then used the population density to redistribute the variable and aggregate to LGA boundaries.

Poverty and extreme poverty maps were computed by summing values based on LGA boundary across the test area. Data related to access to infrastructure and mass media were available as percentages (in the states) and as such were redistributed to LGAs using the population data (weighted distribution).

Number of births (NBTH) and number of pregnant women (NPREG) were available as grid files and were processed the same way as the poverty-related dataset. Population of females (FMLE) (at LGA), population of 14-years-old and below (BLW14) (at state level), population of 65-year-olds and above (ABV65) (at state level) and population of persons with disability (PWDS) (at state level) were all available as percentages and were processed by weighted distribution. The number of dependent young people younger than 15 years was adopted because the age groups available in the dataset are 0–4, 5–14, 15–29 etc., making it impossible to separate the age group 15–18.

Net primary attendance rate (NPAR), net secondary attend-ance rate (NSAR) and literacy rate for 15-year-olds and above (LIT15) were all also available as percentages at state levels and processed using weighted distribution to obtain LGA values. For population density (POPD), grid data were aggregated to the boundaries of LGA. Car ownership (COWN) was computed using the World Bank average reported data (World Bank 2007) (31 cars per 1000 individuals) and weighted by the UN 2010 population estimates (GeoData Institute n.d.). Accessibility (RDACS) was measured with the number of junction counts (Beale 2012) within a GIS platform. This number of junctions was then weighted by area of each LGA.

The state-level dataset was converted to shapefiles (polygon) and then raster. The resulting raster dataset was then resampled using the bilinear interpolation technique and checked using correlation to examine the level of agreement between the original raster data and the resampled raster data. Resampling was carried out to ensure that all raster data have uniform grid size. The result of the correlation check shows that the resampled dataset has a high agreement with the original (*r* ≥ 0.99).

Normalisation was employed to restrict the value of the indicator between a minimum of 0 and maximum of 1. This normalisation process provides a common measurement scale. Two different methods of normalisation were adopted for (1) variables where high values indicate high vulnerability and (1) variables where low value indicate high vulnerability. In case of (1), state and LGA totals were computed, after which the state value was divided by the LGA value (to derive X values). In case of (2), after deriving the state and LGA values, the LGA was subtracted from the state value to obtain X values. Finally, X values obtained were divided by the maximum X value obtained for each state in the study area to derive the SVS.

In order to eliminate redundant variables and identify the dimension within the SVS values, the study adopted the method used by Lawal (2009), that is, a two-staged feature extraction process utilising correlation analysis and factor analysis. Clusters of variables with correlation ≥ 0.9 were collated, and some variables were dropped (Table 2). The negatively correlated variables were retained, because negatively correlated variables will likely show up in the same group but at the opposite ends of the axis when subjected to factor analysis (Lawal 2009). Initial correlation analysis was carried out to eliminate intradomain redundancies whilst the second correlation analysis was carried out to eliminate interdomain redundancies. The results of this operation are presented in the section on feature selection for SoVI computation below.

**Table 2:** Result of redundancy elimination by correlation analysis.

Domains	1st Correlation analysis	2nd Correlation analysis
Socioeconomic status	NPOV; IWAT	NPOV
Household composition and disability	NPREG; FMLE; BLW14; PWDS	FMLE; PWDS
Literacy (Changed from minority status and language)	LIT15	LIT15
Housing and transportation	POPD; RDACS; COWN	POPD; RDACS

NPOV, Poverty; IWAT, improved water; NPREG, Number of pregnant women; FMLE, Population of females; BLW14, 14-year-olds and below; PWDS, persons with disability; LIT15, Literacy rate – 15 and over; POPD, Population density; RDACS, Road density; COWN, Car ownership.

Using an equal weighting method, SVS maps of the extracted subset of variables were summed to generate a SoVI. This was then reclassified such that each LGA falls into one of three classes (using quantile classification), with class 1 as the least vulnerable and class 3 as the most vulnerable.

Errors are inherent in any compound index and a SoVI is no exception. The challenge is how to effectively quantify this error. For this study, each source of information used has its own level of uncertainty and error; some were stated by the sources and others were not. Therefore, methods and techniques for computation of this error need to be devised. For this study, we adopted confidence interval and margin of error as indicators of uncertainty of the SVS computed. Developing approaches to quantifying error for this index is ongoing.

## Results and discussion

An exploration of the SVS values for the 19 variables was carried out using descriptive statistics (mean and standard deviation) for each of the states in this zone. The discussion will be structured according to the domain of the input variables.

### Minority status or language: Literacy

**Figure 3:**
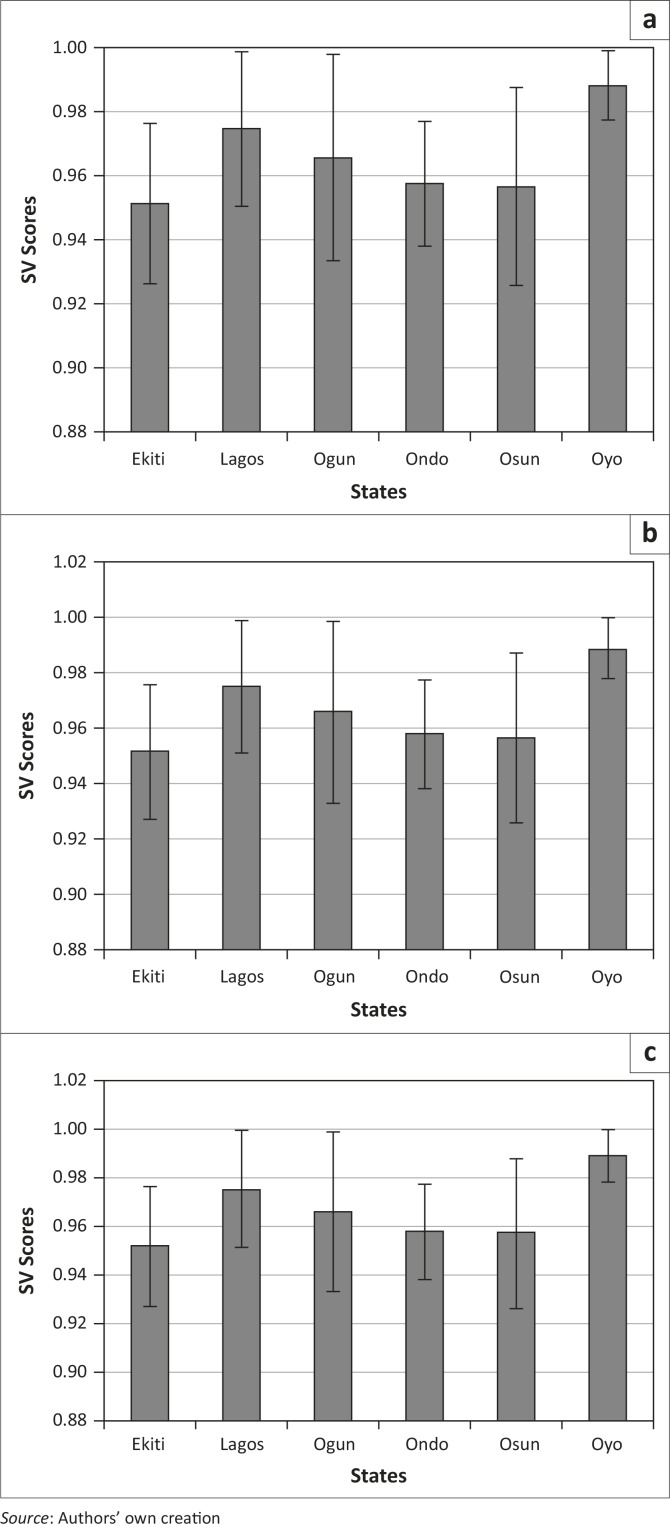
State-level mean and standard deviations of social vulnerability scores for language/literacy-related variables: (a) Net primary attendance rate, (b) Literacy rate – 15 and over and (c) Net secondary attendance rate.

Three variables, namely NPAR, NSAR and LIT15, were examined to represent literacy (Table 1). Examination of the mean SVS (NPAR) revealed that Oyo is the most vulnerable whilst Ekiti is the least vulnerable (Figure 3). NSAR showed the same trend, confirmed by LIT15.

The result shows that there is high literacy across the regions. However, when LGAs are compared there are differences within states, that is, some LGAs are more vulnerable than others. For example, in the case of LIT15, the LGAs Ado-Ekiti (Ekiti), Ifo (Ogun), Ado Odo/Ota (Osun) and Ife North (Osun) have a considerably lower vulnerability compared to their counterparts within the state.

In the region, Oyo is worst off, with high vulnerability scores for literacy. A handful of LGAs across other states also recorded high vulnerability for literacy, but Oyo has a disproportionately large number of relatively highly vulnerable LGAs.

### Socioeconomic status

The number of people in poverty (NPOV) and extreme poverty (EPOV), with access to improved sanitation (ISAN) and improved water (IWAT), with radio (RADP), television (TVP) and electricity (ELECH) in the household were used to describe the socioeconomic vulnerability of the LGAs across the SWGPZ.

**Figure 4:**
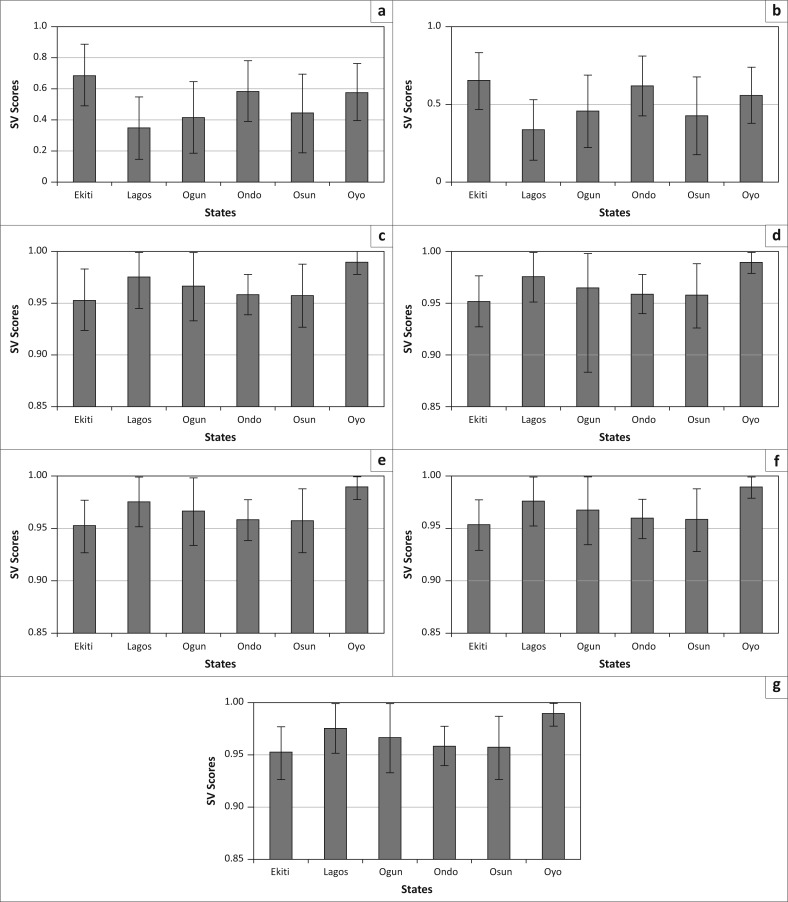
State-level mean and standard deviations of social vulnerability scores for variables related to socioeconomic status: (a) poverty, (b) extreme poverty, (c) access to improved sanitation, (d) access to improved water, (e) radio in household, (f) television in household and (g) electricity in household.

Mean SVS (Figure 4) show similarity across NPOV and EPOV for the SWGPZ. Lagos recorded the lowest mean SVS for both EPOV and NPOV. Osun and Ogun appear to be similar, with SVS slightly higher than that of Lagos (but much better that the remaining states). Ekiti and Ondo recorded the highest mean SVS for EPOV (Ekiti>Ondo>Oyo>Ogun>Osun>Lagos). Mean SVS for NPOV displayed as similar trend as observed for EPOV.

Furthermore, the variation in NPOV and EPOV within the states (represented by the standard deviation bars), shows that Osun is the most heterogeneous state, followed by Ogun. The least heterogeneous state is Oyo, whilst the remaining states are more or less similar. The trend in variation shows that Osun>Ogun>Lagos>Ondo>Ekiti>Oyo in the case of EPOV. For NPOV the trend was found to be slightly different: Osun>Ogun>Lagos>Ekiti>Ondo>Oyo.

Access to facilities such as water, sanitation, electricity and mass media (radio and television) represented another dimension of socioeconomic status. In the case of TVP, RADP, IWAT, ISAN and ELECH, the highest mean SVS were recorded in Oyo (Oyo>Lagos>Ogun>Ondo>Osun>Ekiti). Variation within the state was found to be highest for Ogun. Variations in these variables’ SVS within states showed the trend Ogun>Osun>Ekiti>Lagos>Ondo>Oyo.

Evidently, socioeconomic circumstances of communities are very important in disaster risk management. Evaluation of the socioeconomic characteristics of the SWGPZ shows that Ekiti is the most vulnerable and Lagos is the least vulnerable amongst the six states. The results also indicated that there is a slightly higher heterogeneity common across variables within the domain for Oyo and Lagos. Essentially, vulnerability is quite variable amongst LGAs in Oyo and Lagos compared to other states.

### Household composition and disability

**Figure 5:**
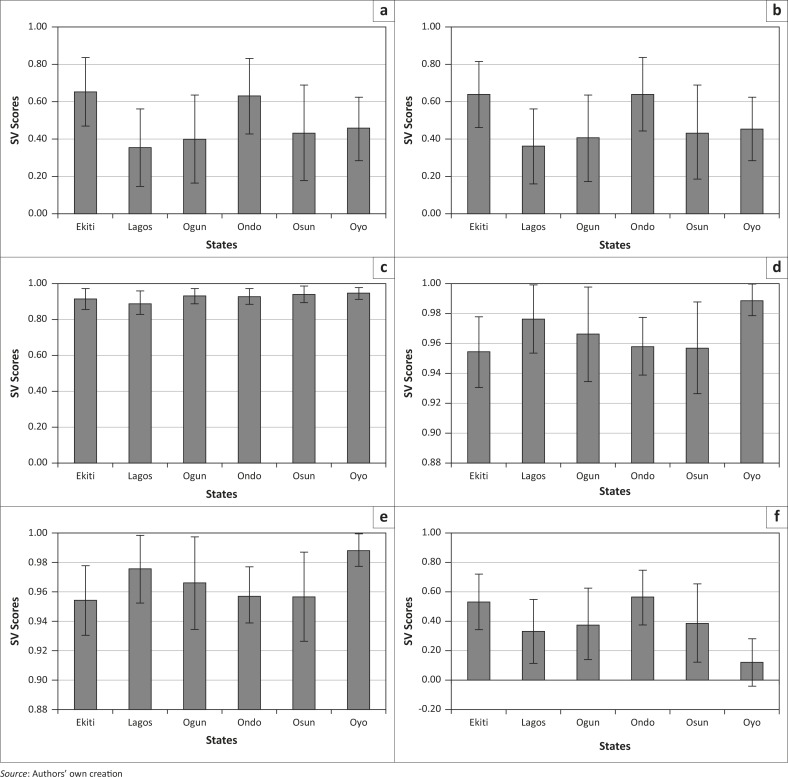
State-level mean and standard deviations of social vulnerability scores for variables related to household composition and disability: (a) number of births, (b) number of pregnant women, (c) population of females, (d) population of 14-year-olds and below, (e) population of 65-year-olds and above and (f) population of persons with disability.

Ekiti and Ondo have the highest mean SVS for NBTH (Ekiti>Ondo>Oyo>Osun>Ogun>Lagos) (Figure 5). In addition, the greatest variation amongst the LGAs was recorded for Osun and Ogun, with Oyo<Ekiti<Ondo<Lagos<Ogun<Osun. Mean SVS for NPREG (Figure 5) showed a trend in which the score for Ondo is slightly greater than for Ekiti (Ondo>Ekiti>Oyo>Osun>Ogun>Lagos). Mean SVS for FMLE are very high, with a trend showing Oyo>Osun>Ogun>Ondo>Ekiti>Lagos. Within the state, Lagos has the most heterogeneous variation and Oyo the least (Lagos>Ekiti>Osun>Ondo>Ogun>Oyo).

Age-related dependent groups BLW14 and ABV65 showed a similarity in pattern of mean SVS at the state level. SVS shows a trend of Oyo>Lagos>Ogun>Ondo>Osun>Ekiti for these two. At the LGA level, Ogun is the most heterogeneous and Oyo the least (Ogun>Osun>Ekiti>Lagos>Ondo>Oyo). More-over, Ogun and Osun have comparatively higher variation amongst their LGAs compared to other states in the region. For PWDS, mean SVS for Ondo is the highest (0.56) whilst Oyo recorded the least (0.12) (Oyo<Lagos<Ogun<Osun<Ekiti<Ondo). At the LGA level, variation in PWDS SVS was found to be highest in Osun with a trend across the regions of Osun>Ogun>Lagos>Ekiti>Ondo>Oyo. Variation for Oyo was also found to be greater than the mean of this variable. This can be attributed to the influence of a few very high values.

### Housing and transportation

**Figure 6:**
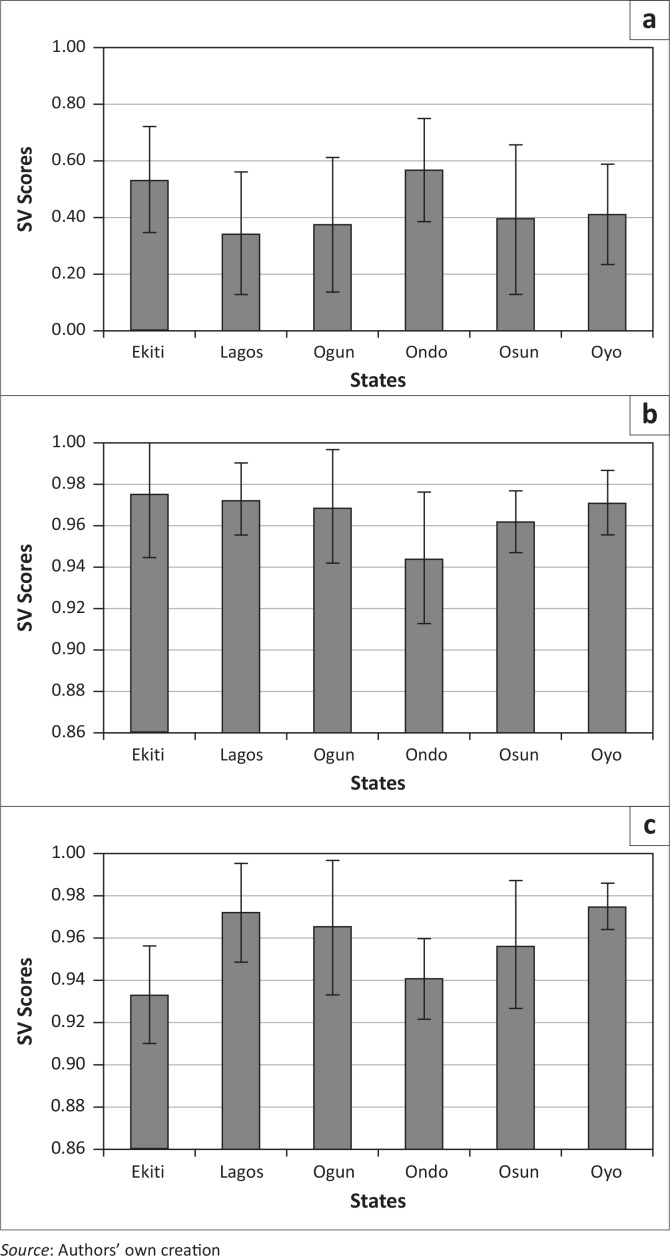
State-level mean and standard deviations of social vulnerability scores for variables related to housing and transportation: (a) population density, (b) road density and (c) car ownership.

In the case of POD (used to indicate crowding), Ondo was found to have the highest mean (0.57) and Lagos the lowest (0.34) (Ondo>Ekiti>Oyo>Osun>Ogun>Lagos). Within the state, the highest variation was recorded for Osun, followed by Ogun, whilst Oyo (Figure 6) was found to be the most homogeneous (Osun>Ogun>Lagos>Ekiti>Ondo>Oyo).

For COWN, most of the states have high mean SVS values (Figure 6). Oyo had the highest mean values, whilst Ekiti had the lowest. Variability amongst the LGAs within the states was highest in Ogun and Osun whilst the least heterogeneous state was Oyo.

Mean SVS for RDACS were high across the region and displayed the trend Ondo<Osun<Ogun<Oyo<Lagos<Ekiti. At the LGA level, Ondo had the highest variation amongst the LGAs and Oyo has the lowest.

### Feature selection for social vulnerability index computation

The first correlation analysis reduced 19 variables to 10 (Table 2). In a subsequent correlation analysis, COWN versus. LIT15, IWAT, BLW14 and POPD versus. NPREG variable pairs displayed above-threshold, positive correlation and IWAT, BLW14, NPREG and COWN were dropped, leaving only six to be subjected to factor analysis.

Factor analysis was carried out to group these six variables so that the correlation within groups is large and the correlation between groups is small, thus maximising variance within groups and minimising variance between groups. Using the correlation matrix of the final six variables as inputs for the factor analysis, a number of identified factors were chosen based on eigen values and cumulative proportion of explained variance by including another factor (group). After factoring the six representative variables using the principal component factor analysis, two factors were identified and together were found to explain about 75% of the variation across the SVS dataset. Based on the eigen values in Table 3, it is evident that the two factors met the Kaiser criterion (Kaiser 1960) for retaining factors.

**Table 3:** Results of factor analysis of selected variables.

Statistics	Factor 1	Factor 2
Eigenvalues	3.449	1.062
Cum. variance	57.481	17.695
**Variables**	**Factor loading after Varimax rotation**	
RDACS	−0.119	0.909
PWDS	0.869	−0.135
FMLE	−0.591	−0.4
NPOV	0.818	0.19
POPD	0.895	−0.099
LIT15	−0.927	0.103

Cum., cumulative; RDACS, Road density; PWDS, persons with disability; FMLE, Population of females; NPOV, Poverty; POPD, Population density; LIT15, Literacy rate – 15 and over.

Five of the six variables showed high loading into the two factors (i.e. absolute loading value > 0.7). As FMLE had an absolute factor loading of about 0.6, it was acceptable to put this variable in factor 1. RDACS was the only variable loading highly into factor 2 and was therefore designated ‘accessibility’. Based on the range of variables included in factor 1, this factor was designated ‘socioeconomic conditions’.

**Figure 7:**
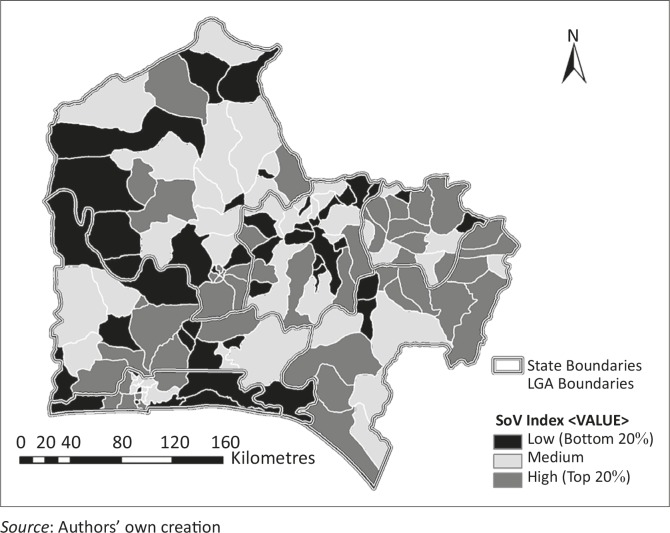
Spatial distribution of social vulnerability index classes across the South West Geopolitical Zone of Nigeria.

Using the six variables identified, an equal weight summation was carried out within GIS to compute the SoVI for the SWGPZ. The result was reclassified using quantile classification (Figure 7). Across the SWGPZ, about 47 LGAs had a relatively low vulnerability on the SoVI scale, whilst 42 and 48 LGAs belonged to the medium and high vulnerability classes respectively.

Within the states, analysis of the SoVI showed that 61% of LGAs in Ondo and 56% of LGAs in Ekiti belongs to the highly vulnerable class. About 50% of the LGAs in Osun and Ogun belongs to the low vulnerability class. Lagos and Oyo have a balanced (relatively equal) distribution of LGAs across the three classes of vulnerability.

Generally, the distribution of vulnerable LGAs shows a varying pattern across the SWGPZ. This variation could be attributed to the underlying demographic and socioeconomic peculiarities of each state. Thus, when these variables are summed up, it becomes apparent where socially vulnerable people are located across the region and within each state.

### Error representation

Using a 95% confidence interval, the margin of error for each of the selected inputs for the SoVI was computed. For NPOV the margin of error is greatest for Ekiti whilst Oyo recorded the lowest margin of error for this variable (Table 4). For FMLE, the margin of error ranges between ± 0.010 and ± 0.026, with Lagos recording the highest and Oyo the lowest. Ogun recorded the highest margin of error for PWDS, LIT15 and POPD, whilst Oyo recorded the lowest for PWDS and LIT15. Osun has the lowest margin of error for POPD, whilst both Oyo and Osun have low margins of error for accessibility (indicated by RDACS).

**Table 4:** Mean social vulnerability scores and margin of error for social vulnerability index components at 95% confidence interval.

SoVI components	NPOV	FMLE	PWDS	LIT15	POPD	RDACS
Ekiti	0.689 ± 0.113	0.918 ± 0.024	0.531 ± 0.109	0.952 ± 0.015	0.535 ± 0.109	0.975 ± 0.016
Lagos	0.348 ± 0.104	0.893 ± 0.026	0.333 ± 0.099	0.975 ± 0.011	0.340 ± 0.097	0.973 ± 0.013
Ogun	0.417 ± 0.111	0.933 ± 0.020	0.382 ± 0.120	0.966 ± 0.016	0.374 ± 0.118	0.969 ± 0.012
Ondo	0.587 ± 0.097	0.928 ± 0.020	0.563 ± 0.106	0.958 ± 0.011	0.569 ± 0.105	0.944 ± 0.016
Osun	0.442 ± 0.078	0.943 ± 0.014	0.392 ± 0.078	0.957 ± 0.009	0.391 ± 0.078	0.962 ± 0.006
Oyo	0.577 ± 0.069	0.948 ± 0.010	0.124 ± 0.057	0.989 ± 0.005	0.413 ± 0.083	0.971 ± 0.006

SoVI, social vulnerability index; NPOV, poverty; FMLE, population of females; PWDS, population of persons with disability; LIT15, literacy rate – 15 and over; POPD, population density; RDACS, road density.

The margin of error values gave us an indication of how well the estimates represented the LGAs. It was revealed that relative to their means, uncertainty across the states was highest for PWDS, POPD and NPOV.

## Conclusion

The results of the study represent an important dimension in the study of disaster risk management in Nigeria. Currently there is a dearth of research on the mapping of social vulnerability. The result complements disaster management initiatives across the country and decision making authorities. Furthermore, the study provides an opportunity for the development of spatial database infrastructure for monitoring people and places for disaster risk management in the country.

In the collation of relevant variables, the study explored 19 variables, covering aspects such as socioeconomic status, household composition, disability, literacy and housing. The exploration of these variables revealed the extent of variability amongst the LGAs within each state and the SWGPZ. Feature identification using correlation and factor analysis revealed that there are six important variables within this subset which can be grouped into two dimensions, namely accessibility and socioeconomic conditions.

The concept of SoVI development illustrated in this study shows some of the challenges with regard to modelling in an environment where data collection is problematic. However, it shows that with the integration of GIS, data can be collated and modelled to answer important questions, such as where vulnerable groups are located. As can be seen from the results, there are noticeable differences amongst the LGAs in the SWGPZ. Furthermore, the model makes it possible to identify LGAs with higher levels of vulnerability in comparison to other LGAs within their respective states.

The SoVI showed that Ondo and Ekiti have more vulnerable LGAs (by percentage) than other states in the SWGPZ whilst about half of the LGAs in Osun and Ogun belong to the least socially vulnerable class. The distribution shows that there are great differences within state as well as across the SWGPZ. This necessitates the need for continuous monitoring to study the evolution and dynamics of social vulnerability in the country. This will help in the implementation of effective disaster risk management plans. Implementing modelling within GIS will also aid the dissemination of maps produced, thereby facilitating access to the information by policy makers, researchers and other stakeholders.

Factors responsible for social vulnerability differ from region to region and place to place, as does the importance of each factor when used in modelling. Thus the model constructed using equal weight in the computation of the SoVI may not adequately represent the full extent of spatial variation in social vulnerability across the country. Furthermore, the availability and quality of data can influence the validity of the index. Moreover, there is a need to continuously improve the index as relevant and up to date data become available and to compare the index outcome with impact in order to ascertain the validity of this model.
